# Prediction of in-hospital stroke mortality in critical care unit

**DOI:** 10.1186/s40064-016-2687-2

**Published:** 2016-07-11

**Authors:** Wei-Min Ho, Jr-Rung Lin, Hui-Hsuan Wang, Chia-Wei Liou, Ku-Chou Chang, Jiann-Der Lee, Tsung-Yi Peng, Jen-Tsung Yang, Yeu-Jhy Chang, Chien-Hung Chang, Tsong-Hai Lee

**Affiliations:** Dementia Center and Department of Neurology, Linkou Medical Center, Chang Gung Memorial Hospital, No.5, Fuxing St., Guishan Dist., Taoyuan City, 333 Taiwan, ROC; Clinical Informatics and Medical Statistics Research Center, Chang Gung University, No.261, Wenhua 1st Rd., Guishan Dist., Taoyuan City, 333 Taiwan, ROC; Department of Healthcare Management, College of Management, Chang Gung University, No.261, Wenhua 1st Rd., Guishan Dist., Taoyuan City, 333 Taiwan, ROC; Stroke Center and Department of Neurology, Kaohsiung Medical Center, Chang Gung Memorial Hospital, No.123, Dapi Rd., Niaosong Dist., Kaohsiung City, 833 Taiwan, ROC; Stroke Center and Department of Neurology, Chiayi Medical Center, Chang Gung Memorial Hospital, No.6, Sec. W., Jiapu Rd., Puzi City, 613 Chiayi County Taiwan, ROC; Department of Neurology, Chang Gung Memorial Hospital, No.222, Maijin Rd., Anle Dist., Keelung City, 204 Taiwan, ROC; Department of Neurosurgery, Chiayi Medical Center, Chang Gung Memorial Hospital, No.6, Sec. W., Jiapu Rd., Puzi City, 613 Chiayi County Taiwan, ROC; Stroke Center and Department of Neurology, Linkou Medical Center, Chang Gung Memorial Hospital, No.5, Fuxing St., Guishan Dist., Taoyuan City, 333 Taiwan, ROC

**Keywords:** Cerebrovascular disease, Intensive care unit, Outcome, Mortality, Risk prediction

## Abstract

**Background:**

Critical stroke causes high morbidity and mortality. We examined if variables in the early stage of critical stroke could predict in-hospital mortality.

**Methods:**

We recruited 611 ischemic and 805 hemorrhagic stroke patients who were admitted within 24 h after the symptom onset. Data were analyzed with independent t test and Chi square test, and then with multivariate logistic regression analysis.

**Results:**

In ischemic stroke, National Institutes of Health Stroke Scale (NIHSS) score (OR 1.08; 95 % CI 1.06–1.11; *P* < 0.01), white blood cell count (OR 1.11; 95 % CI 1.05–1.18; *P* < 0.01), systolic blood pressure (BP) (OR 0.49; 95 % CI 0.26–0.90; *P* = 0.02) and age (OR 1.03; 95 % CI 1.00–1.05; *P* = 0.03) were associated with in-hospital mortality. In hemorrhagic stroke, NIHSS score (OR 1.12; 95 % CI 1.09–1.14; *P* < 0.01), systolic BP (OR 0.25; 95 % CI 0.15–0.41; *P* < 0.01), heart disease (OR 1.94; 95 % CI 1.11–3.39; *P* = 0.02) and creatinine (OR 1.16; 95 % CI 1.01–1.34; *P* = 0.04) were related to in-hospital mortality. Nomograms using these significant predictors were constructed for easy and quick evaluation of in-hospital mortality.

**Conclusion:**

Variables in acute stroke can predict in-hospital mortality and help decision-making in clinical practice using nomogram.

## Background

Acute stroke requiring intensive care usually causes high mortality, and the subsequent long-term disability places hard burden on healthcare system (Go et al. 2014). The prognosis may be attributed to various comorbid factors and causative mechanisms (Damian et al. 2013; Kiphuth et al. 2010). Some factors, such as overt fluctuation of blood pressure (BP) (Mayer et al. 2011; Tikhonoff et al. 2009), impaired renal function (Muntner et al. 2012; Mahmoodi et al. 2014), leukocytosis (Tokgoz et al. 2014; Kazmierski et al. 2004), and initial stroke severity assessed with National Institutes of Health Stroke Scale (NIHSS) (Fonarow et al. 2012), are reported to be related to worsening stroke symptoms and unfavorable prognosis.

Upon emergent condition, there are limited parameters available for the prediction of in-hospital mortality in critical stroke and for decision-making on the scene. Until now, the risk prediction for critical stroke is rarely discussed, especially for hemorrhagic stroke.

We conducted this multi-center database analysis study to examine whether the combination of initial clinical and laboratory variables was predictive to critical stroke mortality. Nomograms using these significant predictors of ischemic and hemorrhagic strokes are also constructed for easy and quick evaluation of in-hospital mortality.

## Methods

### Population

From January, 2009 to December, 2011, stroke patients admitted to neurology intensive care unit (ICU) from emergency department (ED) were prospectively registered in Stroke Registry in Chang Gung Healthcare System (SRICHS) (Lee et al. 2011) and retrospectively analyzed. The SRICHS is an electronic chart-based stroke registry system which conducts electronic chart recording and data registry simultaneously. This study extracted deidentified data from three branch hospitals located in the northern, middle and southern parts of Taiwan. This study was performed in accordance with the ethical standards laid down in the 1964 Declaration of Helsinki and was approved by the Institutional Review Board of Chang Gung Healthcare System.

**Fig. 1:**
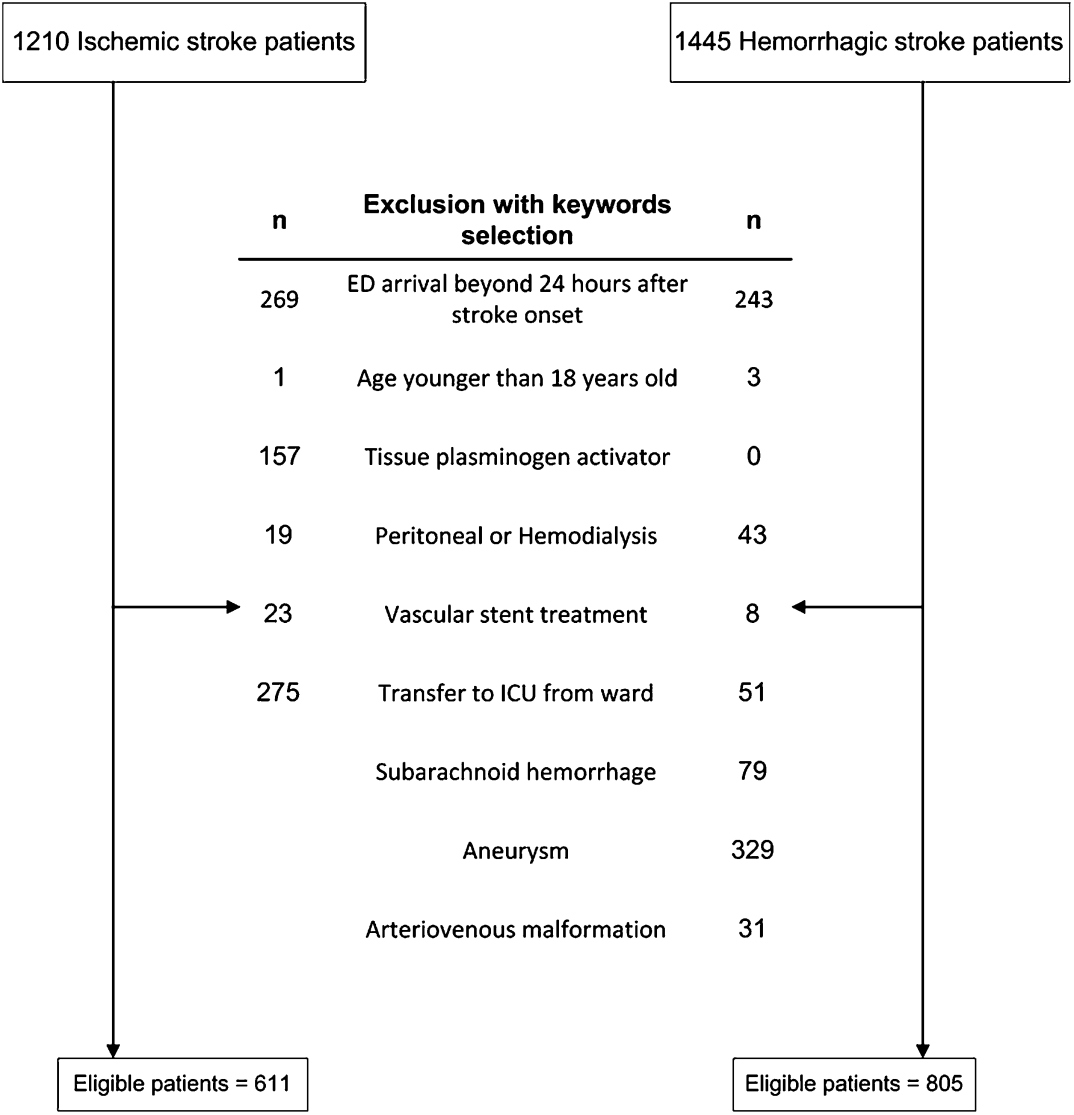
Flow chart of patient recruitment. *ED* emergency department, *ICU* intensive care unit

Acute stroke patients who could not follow order clearly or who were at the risk of stroke progression and cardiopulmonary failure such as large ischemic or hemorrhagic stroke or brain stem ischemic or hemorrhagic stroke were arranged for ICU admission. We aimed to investigate variables that might affect the pathophysiology of acute severe stroke and short-term mortality in critical stroke patients without major comorbidity at stroke onset. Therefore, patients who were under the age of eighteen; attended ED beyond 24 h after stroke onset; underwent peritoneal or hemodialysis; who were admitted to neurology ward with minor stroke initially and then were transferred to ICU due to comorbidity, such as infection or gastrointestinal bleeding were excluded (Fig. 1). Ischemic stroke patients who received recombinant tissue plasminogen activator (rt-PA) or who had intracranial or extracranial vascular stent placement were not included under the consideration that these interventions might alter disease progression. Subarachnoid hemorrhage (SAH), traumatic intracranial hemorrhage (ICH), aneurysm or arteriovenous malformation (AVM) were excluded because of the different mechanisms and treatments from primary hemorrhagic stroke.

### Data collection and variable definition

Upon admission, Glasgow Coma Scale (GCS), NIHSS score and modified Rankin Scale (mRS) were assessed. Vital signs including systolic and diastolic BP, body temperature (BT), and heart rate were measured. The definition of hypertension followed the guideline of the Seventh Report of the Joint National Committee on Prevention, Detection, Evaluation, and Treatment of High Blood Pressure (JNC 7) (2004). Body mass index (BMI) was calculated by weight in kilograms divided by squared height in meters. Initial laboratory data including white blood cell (WBC) and differential count, hemoglobin, platelet count, creatinine (Cr), blood urea nitrogen (BUN) and electrolytes were collected upon patient’s arrival to ED. Glomerular filtration rate (GFR) was calculated according to the Modification of Diet in Renal Disease (MDRD) study equations (Stevens et al. 2006). Histories of comorbidities and risk factors including previous stroke, heart disease (Including heart failure, and arrhythmic, valvular and ischemic heart diseases), hypertension, diabetes mellitus (DM), dyslipidemia, dialysis, cancer, cigarette or alcohol consumption were recorded. The treatment of stroke followed the guidelines of Taiwan stroke society (Chang et al. 2008) and American Stroke Association (Adams et al. 2007; Broderick et al. 2007). All recruited patients received acute management in ICU and were then transferred to ordinary ward if the clinical status became stable. Mortality was recorded if patients were announced expired during hospitalization.

### Statistical analysis

Variables were checked with Shapiro–Wilk test initially. Numerical variables such as age, BT, systolic BP, diastolic BP, NIHSS score, WBC count, Cr, BUN, BUN and Cr (BUN/Cr) ratio and BMI were presented as mean and standard deviation. Categorical variables such as gender, history of hypertension, DM, dyslipidemia, heart disease and previous stroke were presented as number and percentage. Independent t test and Chi square test were used to examine the significance depending on the type of variables. A two-sided probability value of less than 0.05 was considered significant.

Significant variables from univariate tests were forward selected and used as predictive variables in multivariate logistic regression analysis. Numerical variables were included as linear function. Due to the U-shape characteristic of BPs (Leonardi-Bee et al. 2002; Vemmos et al. 2004), systolic BP was divided into two groups: Group 1 with systolic BPs <100 or >180 mmHg and Group 2 with systolic BPs between 100 and 180 mmHg. The predictive accuracy of the derived model was assessed by discrimination and calibration methods. The receiver operating characteristic curve was drawn and the area under the curve was calculated to obtain concordance statistic (c-statistic) which was a measure of predictive discrimination. Goodness of fit was checked by le Cessie and Houwelingen test to know the fitness of the model. In order to see the level of agreement between predictive and observed values, we generated calibration curves through bootstrapping method. A point system was developed in a range of 0–100 points. The impact of variables to the system was calculated in proportion to their estimated beta coefficients. The variable with the largest beta coefficient was given as 100 points and the rest of the variables were assigned points according to the proportion of their beta coefficients to the largest ones. After the above procedures, a graphical calculating chart was developed. Statistical analysis was performed using R open source software version 3.0.1, and the rms package for logistic regression and nomogram construction.

## Results

A total of 1210 ischemic and 1445 hemorrhagic stroke patients admitted to neurology ICU were recruited from SRICHS. The following patients were excluded including 269 ischemic and 243 hemorrhagic stroke patients with ED arrival time longer than 24 h after stroke onset, 4 with age younger than 18 years old, 62 with peritoneal dialysis or hemodialysis, 31 with vascular stent placement, and 157 ischemic stroke patients with rt-PA treatment. There were 326 patients admitted to ICU from neurology ward due to severe gastrointestinal bleeding, septic shock and respiratory failure, and they were also excluded from analysis. In hemorrhagic stroke group, 79 patients with SAH, 329 with aneurysm and 31 with AVM were excluded (Fig. 1).

Demographic results in univariate analysis, including clinical characteristics and laboratory data, are presented in Table 1. The in-hospital mortality rates of ischemic and hemorrhagic strokes were 15.9 and 20.4 %, respectively. In ischemic stroke group, patients with in-hospital mortality were significantly older than those who survived (69.8 ± 13.5 vs. 75.6 ± 12.9, *P* < 0.01). In hemorrhagic stroke group, patients with mortality had significantly higher pulse rate, lower systolic BP and higher frequency of heart disease than those who survived (*P* < 0.01, *P* = 0.03 and *P* < 0.01, respectively). In both groups, NIHSS score (*P* < 0.01 in both groups), WBC count (*P* = 0.02 in ischemic stroke and *P* < 0.01 in hemorrhagic stroke), BUN (*P* < 0.01 in both groups) and GFR (*P* = 0.01 in ischemic stroke and *P* = 0.03 in hemorrhagic stroke) were significantly higher in mortality group. Furthermore, death patients in hemorrhagic stroke had higher level of Cr (*P* < 0.01), while death patients in ischemic stroke had higher BUN/Cr ratio (*P* < 0.01) than survived patients did.

**Table 1:** Comparison of clinical characteristics between survival and mortality groups in ischemic and hemorrhagic strokes

Variables^a^	Ischemic stroke (n = 611)			Hemorrhagic stroke (n = 805)		
	Survival (n = 514)	Mortality (n = 97)	*P*	Survival (n = 641)	Mortality (n = 164)	*P*
Age (years)	69.8 ± 13.5	75.6 ± 12.9	<0.01	62.0 ± 14. 6	61.9 ± 15.7	0.92
Male, n (%)	304 (59.1)	45 (46.4)	0.03	387 (60.4)	111 (67.7)	0.10
BMI (kg/m^2^)	23.5 ± 4.1	23.1 ± 4.3	0.43	23.8 ± 4.3	23.7 ± 4.6	0.84
NIHSS	16.4 ± 10.1	26.2 ± 10.1	<0.01	17.4 ± 13.3	35.0 ± 9.1	<0.01
Vital signs						
BT (°C)	36.7 ± 0.7	36.8 ± 0.8	0.39	36.8 ± 0.8	37.0 ± 1.5	0.10
Pulse (per min)	83.0 ± 19.6	85.0 ± 19.1	0.36	81.1 ± 16.5	89.0 ± 27.0	<0.01
SBP (mmHg)	147.0 ± 27.4	146.8 ± 35.0	0.96	148.2 ± 27.1	139.0 ± 48.8	0.03
DBP (mmHg)	83.0 ± 16.5	79.1 ± 22.4	0.12	83.6 ± 17.2	79.3 ± 27.3	0.06
Laboratory data						
WBC count (10^9^/L)	9.3 ± 4.5	10.7 ± 5.2	0.02	10.6 ± 4.6	12.0 ± 5.4	<0.01
Creatinine (mg/dL)	1.2 ± 0.9	1.4 ± 1.6	0.21	1.1 ± 1.0	1.7 ± 2.2	<0.01
BUN (mg/dL)	18.1 ± 12.0	24.0 ± 16.1	<0.01	16.8 ± 11.1	24.6 ± 18.4	<0.01
BUN/Cr ratio	16.8 ± 6.9	20.4 ± 10.8	<0.01	17.0 ± 8.4	18.3 ± 9.5	0.15
GFR	77.4 ± 33.6	67.1 ± 31.7	0.01	87.0 ± 35.7	79.1 ± 41.3	0.03
History of comorbidity, n (%)						
Hypertension	386 (75.1)	77 (79.4)	0.44	453 (70.7)	123 (75.0)	0.32
Diabetes mellitus	179 (34.8)	35 (36.1)	0.90	138 (21.5)	46 (28.0)	0.09
Heart disease	227 (44.2)	48 (49.5)	0.39	81 (12.6)	38 (23.2)	<0.01
Dyslipidemia	87 (16.9)	12 (12.4)	0.33	27 (4.2)	9 (5.0)	0.62
Previous stroke	160 (31.1)	34 (35.1)	0.52	94 (14.7)	22 (13.4)	0.78

*BMI* body mass index, *BT* body temperature, *BUN* blood urea nitrogen, *BUN/Cr ratio* BUN/Creatinine ratio, *GFR* glomerular filtration rate according to the Modification of Diet in Renal Disease (MDRD) study equation, *ICU* intensive care unit, *NIHSS* National Institutes of Health Stroke Scale, *SBP* systolic blood pressure, *DBP* diastolic blood pressure, *WBC* white blood cell

^a^Variables are reported as mean ± standard deviation or number (percentage)

**Fig. 2:**
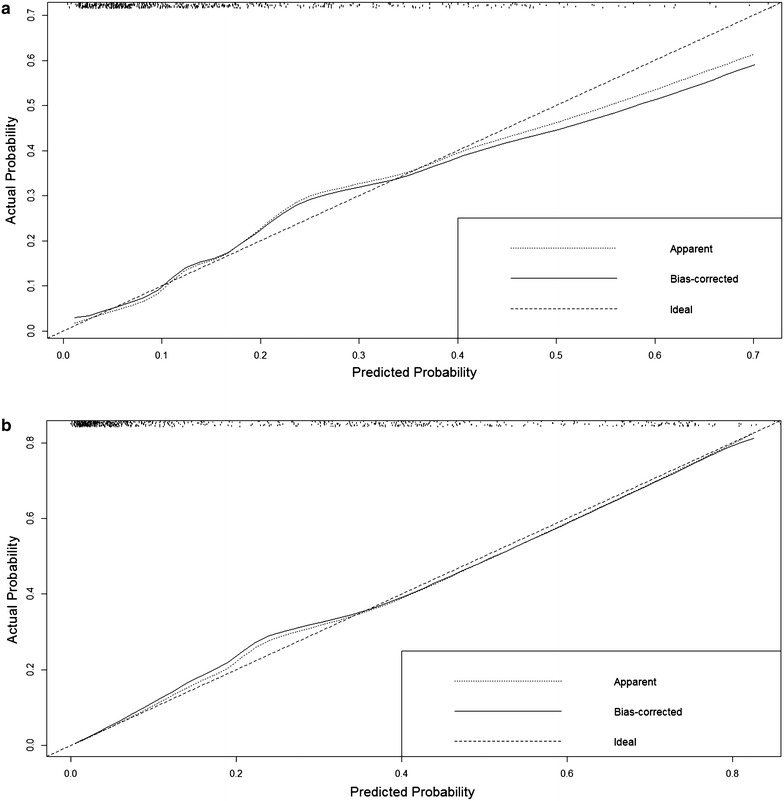
Calibration curves in the prediction model of observed and predicted in-hospital mortality. **a** Calibration curve in ischemic stroke group (Z = 0.65, *P* = 0.52) shows mild overestimation in high risk group, **b** calibration curve in hemorrhagic stroke (Z = 0.87, *P* = 0.36)

**Fig. 3:**
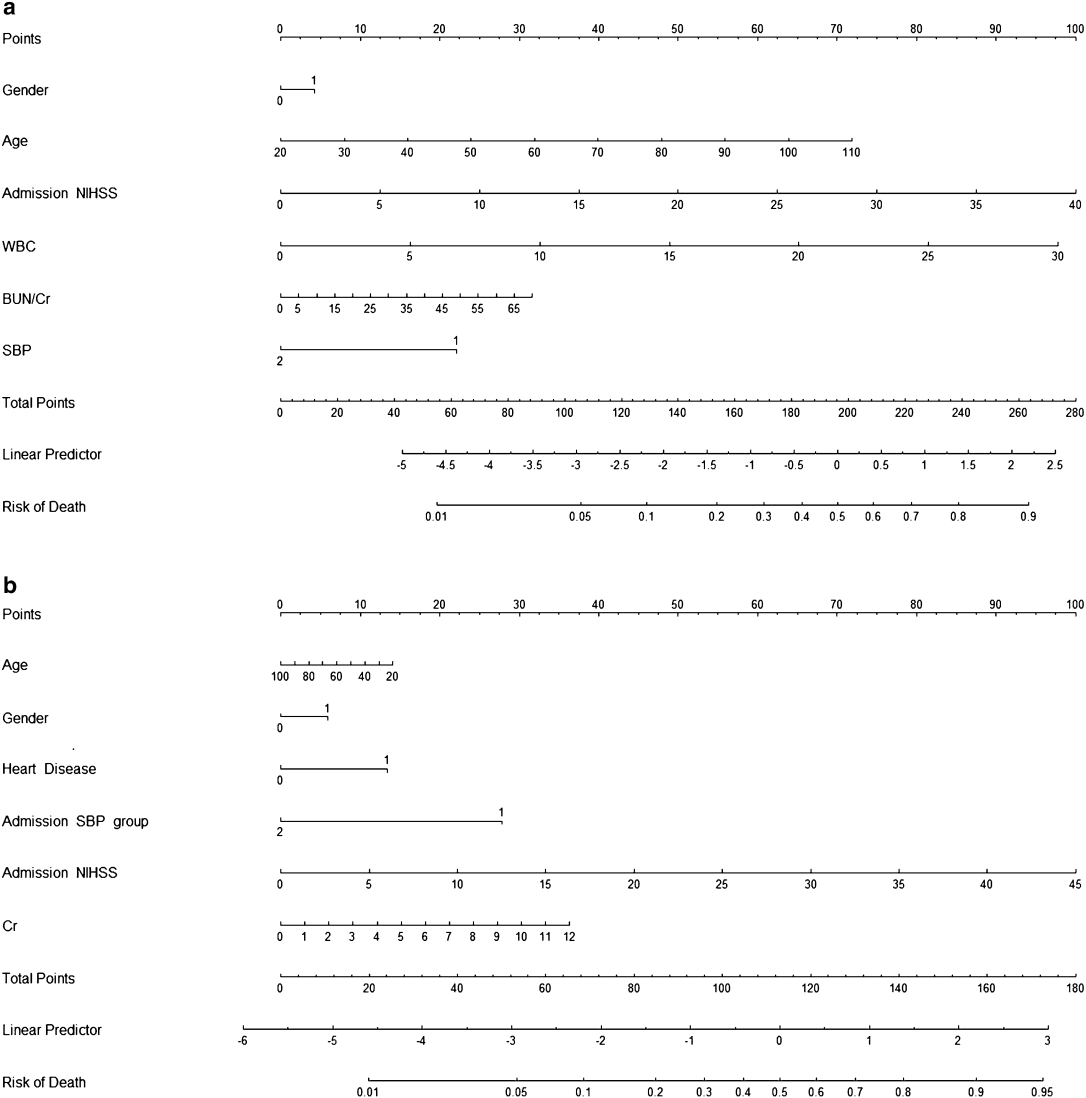
Nomograms for risk prediction of in-hospital mortality in acute stroke. **a** Nomogram for ischemic stroke, **b** nomogram for hemorrhagic stroke. For clinical use of nomogram, physicians first put data onto each scale of variables and then align vertically to the above point axis to get points. Then, sum up the points of each variable to obtain the final score and convert the score into probability of mortality. Due to the U-shape distribution, systolic BP was categorized into Group 1 with systolic BPs <100 or >180 mmHg and Group 2 with systolic BPs between 100 and 180 mmHg. *BUN/Cr ratio* blood urea nitrogen to creatinine ratio, *NIHSS* National Institutes of Health Stroke Scale, *SBP* systolic blood pressure, *WBC* white blood cell

Significant variables in univariate analysis were assigned to build up multivariate logistic regression model. In ischemic stroke group, age, gender, NIHSS score, WBC count, BUN and BUN/Cr ratio were chosen as covariates. The systolic BP was added under the consideration of clinical importance. Multivariate analysis (Table 2) showed NIHSS score (OR 1.08; 95 % CI 1.06–1.11; *P* < 0.01), WBC count (OR 1.11; 95 % CI 1.05–1.18; *P* < 0.01), systolic BP (OR 0.49; 95 % CI 0.26–0.90; *P* = 0.02) and age (OR 1.03; 95 % CI 1.00–1.05; *P* = 0.03) were significantly associated with in-hospital mortality, while BUN/Cr ratio was not. Fitness of the predictive model (Fig. 2a) was well calibrated (Z = 0.65, *P* = 0.52) with mild overestimation in high risk patients. Discriminative examination showed c-statistic = 0.79. In conversion to nomogram (Fig. 3a), NIHSS score was assigned to be 100 points and the rest of the variables were appointed in proportion to their beta coefficients.

**Table 2:** Factors affecting in-hospital mortality in multivariate logistic regression model

	Odds ratio	95 % CI	*P*
Ischemic stroke			
Age	1.03	1.00–1.05	0.03
Gender	1.15	0.65–2.01	0.63
NIHSS	1.08	1.06–1.11	<0.01
Systolic BP	0.49	0.26–0.90	0.02
WBC count	1.11	1.05–1.18	<0.01
Bun/Cr ratio	1.01	0.98–1.05	0.36
Hemorrhagic stroke			
Age	0.99	0.98–1.01	0.27
Gender	1.34	0.83–2.15	0.23
NIHSS	1.12	1.09–1.14	<0.01
Systolic BP	0.25	0.15–0.41	<0.01
Heart disease history	1.94	1.11–3.39	0.02
Cr	1.16	1.01–1.34	0.04

*BP* blood pressure, *BUN/Cr ratio* blood urea nitrogen to creatinine ratio, *CI* confidence interval, *NIHSS* National Institutes of Health Stroke Scale, *WBC* white blood cell

In hemorrhagic stroke group, NIHSS score, pulse rate, heart disease history, WBC count, systolic BP, BUN and Cr were included into multivariate logistic regression model as predictors. Age and gender were included as general variables for adjustment. In multivariate analysis (Table 2), NIHSS score (OR 1.12; 95 % CI 1.09–1.14; *P* < 0.01), systolic BP (OR 0.25; 95 % CI 0.15–0.41; *P* < 0.01), heart disease history (OR 1.94; 95 % CI 1.11–3.39; *P* = 0.02) and Cr (OR 1.16; 95 % CI 1.01–1.34; *P* = 0.04) were related to in-hospital mortality. WBC count was not significant in multivariate analysis and was excluded from nomogram. The prediction model in hemorrhagic stroke group (Fig. 2b) revealed a good discrimination ability (c-statistic = 0.87). Model fitness was significant (Z = 0.87, *P* = 0.36) and calibration curve showed a steady distribution which means well estimation of agreement between predicted probabilities and observed proportions. Nomogram for hemorrhagic stroke (Fig. 3a) was built according to beta coefficients of the variables.

## Discussion

This retrospective study analyzed variables from patients in acute critical stroke stage and subsequently developed multivariate risk prediction models and nomograms for the aid of decision making in acute stage. Researchers have been trying to come up with stroke risk prediction models for functional decline and mortality. Most prediction models were based on general stroke population, that is to say, recruiting both minor and severe stroke patients (Ntaios et al. 2012; Saposnik et al. 2011; Smith et al. 2010; O’Donnell et al. 2012), and seldom discussed the influence of physiological changes in acute stroke stage.

The derivation of a risk prediction model usually starts from univariate analysis with the selection of significant risk factors from the studied population and then applies these variables to multivariate analysis. Stroke severity, despite of various definitions, was introduced in many models. Our study used NIHSS score as measurement of stroke severity. NIHSS score has been validated to be predictive and discriminative not only in short term stroke mortality but also in long term functional decline (Fonarow et al. 2012; Dhamoon et al. 2012). On initial stroke presentation, NIHSS score was usually higher in hemorrhagic stroke than that in ischemic stroke (Smith et al. 2013) and could predict mortality of hemorrhagic stroke as well (Koivunen et al. 2014). In the widely applied Acute Physiology and Chronic Health Evaluation (APACHE) score, Glasgow Coma Scale is the item representative of nervous system. In one study using APACHE II score to predict stroke mortality in ICU (Moon et al. 2015), the discrimination (c-statistic: 0.77 in ischemic stroke and 0.81 in hemorrhagic stroke) and calibration (*P* = 0.11 in ischemic stroke and *P* = 0.78 in hemorrhagic stroke) outweighed the results assessed with NIHSS solely. Our model, however, using NIHSS and significant covariates, showed a better performance in assessing the model’s accuracy. The difference might be due to the addictive effects from significant covariates in our patient population. A similar effect was found in Get With The Guidelines (GWTG)-Stroke Program Risk Model which showed that the discrimination was improved after the model included NIHSS score as covariate (Smith et al. 2010).

Large strokes in itself or the subsequent diffuse brain edema, hemorrhagic transformation and herniation may cause hemodynamic change if autonomic nuclei or related pathways were compromised (Dutsch et al. 2007; Nagai et al. 2010). Overt fluctuation of BP and wide heart rate variability in acute stroke were reported with poor outcome (Weimar et al. 2010; Gujjar et al. 2004). Heart diseases are causative or comorbid factors to stroke (Jauch et al. 2013) and heart disease history in our hemorrhagic stroke patients was significantly related to in-hospital mortality and provided high impact in multivariate model. Interestingly, although patients with infection were excluded from this study, WBC count was significantly elevated in both groups. Reactive leukocytosis could be seen as an inflammatory response after acute stroke and was related to mortality (Zia et al. 2012). Yet in our multivariate model, WBC count was significant only in ischemic stroke and contributed relatively small impact on hemorrhagic stroke. Deteriorated renal function has been noted in acute stroke, however, there is no conclusion with regard to which biomarker of renal function best predicts the outcome of acute stroke. BUN/Cr ratio, GFR and Cr level had all been reported to relate to mortality in acute stroke (Porter et al. 2010; Liu et al. 2014; Khatri et al. 2014). However, when we further tested BUN/Cr ratio and Cr in our multivariate model, BUN/Cr ratio fits the model better in ischemic stroke and Cr in hemorrhagic stroke. Decreased GFR only related to mortality in ischemic stroke and had relatively small impact in multivariate model.

In contrast to most of the stroke prediction models, which were generated from community-based population or from both minor and severe stroke patients, the present study focused on acute critical stroke patients who needed ICU care and we developed risk prediction models for guidance on clinical decision and management. The GWTG-Stroke Program Risk Model included ischemic stroke patients and used NIHSS, history of risk factors, admission and arrival mode as predictive variables (Smith et al. 2010). The discriminative ability was good (c-statistic = 0.84), however, deviation was noted in their validation sample (*P* < 0.001 in the Hosmer–Lemeshow test). Our result in ischemic stroke patients showed good calibration (*P* = 0.52) but the discriminative ability was in medium level (c-statistic = 0.79). This result may be due to the critical condition of our patient population, and it was known that higher disease severity may contribute to lower discriminative accuracy (Moons et al. 1997). The prediction model for in-hospital mortality in hemorrhagic stroke is rarely mentioned before, and our study also developed a nomogram for early decision-making in hemorrhagic stroke. We converted multivariate models derived from this study into nomograms for ischemic and hemorrhagic stroke, respectively. These nomograms are intuitively operated tools that are convenient for clinical use (Fig. 3a, b). With increasing expenditure in critical care system, we believe a simple and useful risk prediction model that determinates stroke outcome can help physicians in clinical management and reduce medical expense.

There are some limitations that might affect the interpretation of our results. First, we excluded patients with SAH and cerebral vascular abnormalities, such as aneurysm or AVM. Because these patients received various interventions (angiography, intracranial stent or coil, craniotomy) which could be the major determinant of the in-hospital mortality, and prediction of this disease spectrum might be different from the others. Second, patients who received rt-PA were not included, because rt-PA may alter the stroke progression and therefore the stroke outcome. Third, patient group with limited ethnicity or geographical area has been a concern in developing prediction models (Rempe 2014), external validation, especially in diverse populations, is the future work for this prediction model.

## Conclusion

This work demonstrated that using routine and easily available variables in the acute stage of critical stroke can predict the clinical outcome of both ischemic and hemorrhagic strokes. The nomograms may help physicians in risk prediction of in-hospital mortality.
